# Machine Learning Analyses on Data including Essential Oil Chemical Composition and In Vitro Experimental Antibiofilm Activities against *Staphylococcus* Species

**DOI:** 10.3390/molecules24050890

**Published:** 2019-03-03

**Authors:** Alexandros Patsilinakos, Marco Artini, Rosanna Papa, Manuela Sabatino, Mijat Božović, Stefania Garzoli, Gianluca Vrenna, Raissa Buzzi, Stefano Manfredini, Laura Selan, Rino Ragno

**Affiliations:** 1 Rome Center for Molecular Design, Department of Drug Chemistry and Technology, Sapienza University, P.le Aldo Moro 5, 00185 Rome, Italy; alexandros.patsilinakos@uniroma1.it; 2 Alchemical Dynamics s.r.l., 00125 Rome, Italy; manuela.sabatino@uniroma1.it; 3 Department of Public Health and Infectious Diseases, Sapienza University, P.le Aldo Moro 5, 00185 Rome, Italy; marco.artini@uniroma1.it (M.A.); rosanna.papa@uniroma1.it (R.P.); gianluca.vrenna@uniroma1.it (G.V.); 4 Faculty of Natural Sciences and Mathematics, University of Montenegro, 81000 Podgorica, Montenegro; mijat.bozovic@uniroma1.it; 5 Department of Drug Chemistry and Technology, Sapienza University, P.le Aldo Moro 5, 00185 Rome, Italy; stefania.garzoli@uniroma1.it; 6 Master Course in Cosmetic Sciences, Department of Life Sciences and Biotechnology, University of Ferrara, 44100 Ferrara, Italy; raissa.buzzi@unife.it

**Keywords:** biofilm, *Staphylococcus* species, essential oil, machine learning, antimicrobial

## Abstract

Biofilm resistance to antimicrobials is a complex phenomenon, driven not only by genetic mutation induced resistance, but also by means of increased microbial cell density that supports horizontal gene transfer across cells. The prevention of biofilm formation and the treatment of existing biofilms is currently a difficult challenge; therefore, the discovery of new multi-targeted or combinatorial therapies is growing. The development of anti-biofilm agents is considered of major interest and represents a key strategy as non-biocidal molecules are highly valuable to avoid the rapid appearance of escape mutants. Among bacteria, staphylococci are predominant causes of biofilm-associated infections. Staphylococci, especially *Staphylococcus aureus* (*S. aureus*) is an extraordinarily versatile pathogen that can survive in hostile environmental conditions, colonize mucous membranes and skin, and can cause severe, non-purulent, toxin-mediated diseases or invasive pyogenic infections in humans. *Staphylococcus epidermidis* (*S. epidermidis*) has also emerged as an important opportunistic pathogen in infections associated with medical devices (such as urinary and intravascular catheters, orthopaedic implants, etc.), causing approximately from 30% to 43% of joint prosthesis infections. The scientific community is continuously looking for new agents endowed of anti-biofilm capabilities to fight *S. aureus* and *S epidermidis* infections. Interestingly, several reports indicated in vitro efficacy of non-biocidal essential oils (EOs) as promising treatment to reduce bacterial biofilm production and prevent the inducing of drug resistance. In this report were analyzed 89 EOs with the objective of investigating their ability to modulate bacterial biofilm production of different *S. aureus* and *S. epidermidis* strains. Results showed the assayed EOs to modulated the biofilm production with unpredictable results for each strain. In particular, many EOs acted mainly as biofilm inhibitors in the case of *S. epidermidis* strains, while for *S. aureus* strains, EOs induced either no effect or stimulate biofilm production. In order to elucidate the obtained experimental results, machine learning (ML) algorithms were applied to the EOs’ chemical compositions and the determined associated anti-biofilm potencies. Statistically robust ML models were developed, and their analysis in term of feature importance and partial dependence plots led to indicating those chemical components mainly responsible for biofilm production, inhibition or stimulation for each studied strain, respectively.

## 1. Introduction

A biofilm is a microbially derived sessile community characterized by cells irreversibly attached to a substrate or interface or to each other, embedded in a self-produced matrix of extracellular polymeric substances, which exhibits an altered phenotype with regard to growth, gene expression and protein production [1]. Biofilm resistance to antimicrobials [2] is a complex phenomenon, driven not only by genetic mutation induced resistance, but also by means of increased microbial cell density that supports resistance by means of horizontal gene transfer across cells [3]. Indeed, other mechanisms are involved, such as: (i) low penetration of antimicrobial agents due to the barrier function exerted by the biofilm matrix, (ii) presence of cells exhibiting a high multidrug tolerance, (iii) reduced susceptibility to antibiotics as a consequence of stress adaptive responses or changes in the chemical biofilm microenvironment [4]. The strategies adopted to treat these challenging infections are rapidly changing due to the increasing understanding of biofilm structure and functions. Nonetheless, the prevention of biofilm formation and the treatment of existing biofilms is currently a difficult challenge; therefore, the discovery of new multi-targeted or combinatorial therapies is increasingly urgent [5]. 

The development of anti-biofilm agents is therefore considered of major interest and represents an important strategy since non-biocidal molecules to avoid the rapid appearance of resistant mutants are highly valuable. Among bacteria, staphylococci are prevalent causes of biofilm-associated infections [6]. In particular, *Staphylococcus aureus* (*S. aureus*) is an opportunistic pathogen that can cause serious diseases in humans, ranging from skin and soft tissue infections to invasive infections of the bloodstream, heart, lungs and other organs [7]. In 2013, Nicholson et al. reported that 30% of U.S. population was colonized by *S. aureus* while 1.5% was found to be a carrier of methicillin-resistant *S. aureus* (MRSA), a major cause of healthcare-related infections responsible for a significant proportion of nosocomial infections worldwide. Recently in the U.S. deaths from MRSA infections have exceeded those from many other infectious diseases, including HIV/AIDS [8]. *Staphylococcus epidermidis* (*S. epidermidis*), conventionally considered a commensal of human skin, can cause significant problems when breaching the epithelial barrier, especially during biofilm-associated infection of indwelling medical devices [9,10]. Most diseases caused by *S. epidermidis* exhibit a chronic profile and occur as device-related infections (such as intravascular catheter or prosthetic joint infections) and/or their complications [10]. In view of the above scenario the scientific community is seeking for new agents endowed of anti-biofilm capabilities to fight *S. aureus* and *S epidermidis* infections. Recently, several reports indicated in vitro efficacy of non-biocidal essential oils (EOs) as promising treatment to reduce bacterial biofilm production and prevent the inducing of drug resistance [11]. In different applications, EOs have been found of some efficacy in reducing biofilm production of either *S. aureus* standard strains or MRSA [12,13,14,15,16,17]. In other reports, EOs and some of their purified chemical components have also been proved to inhibit *S epidermidis* biofilm production [18,19,20]. 

Recently machine learning (ML) has been proved as a tool able to deeply investigate the modulatory role of EOs’ chemical components on *Pseudomonas aeruginosa* biofilm production [21,22,23,24]. In particular, 89 EOs extracted in different periods and times of extractions from three different plants were analyzed: 13 EOs (RSEOs) from *Ridolfia segetum Moris* (RS); 32 EOs (FVEOs) from *Foeniculum vulgare* Miller (FV) and 44 EOs (CGEOs) from *Calamintha nepeta* (L.) Savi subsp. *glandulosa* (Req.) Ball, (CG) [22]. In line with that study and with the objective of investigating EOs’ ability to also reduce bacterial biofilm production in other bacteria, herein is reported an extensive study of the 89 EOs samples as potential antibacterial and anti-biofilm agents against *S. aureus* ATCC 6538P, *S. aureus* ATCC 25923, *S. epidermidis* RP62A and *S. epidermidis* O-47. To this purpose, like previously reported [22], ML algorithms were applied to the EOs’ chemical compositions and the determined associated anti-biofilm potencies, with the purpose of shedding light on those components likely mainly responsible for either positive or negative modulation of biofilm production.

## 2. Results

### 2.1. Antimicrobials Activity of EOs

Some FVEOs and CGEOs samples showed MICs at the highest used concentration (Table 1 and Table 2). No antimicrobial activity on staphylococci was recorded for any of the RSEOs, except for the R30 sample that showed a MIC value of 25 mg/mL (Table 3). Only seven out 89 EOs displayed MIC values of 6.25 mg/mL against the two *S. aureus* strains (Table 1, Table 2 and Table 3). As control, MIC was also evaluated for conventional a antibiotic, ofloxacin, belonging to the fluoroquinolone family.

### 2.2. Biofilm Production Modulation by EOs at Selected Fixed Concentrations

Preliminarily, the same representative EOs (2 RSEOs, 3 CGEOs and 3 FVEOs) among the reported 89 used on *P. aeruginosa* [22] were selected, to evaluate the anti-biofilm potency at different concentrations starting from 25 mg/mL, using scalar dilutions (data not shown).

**Figure 1:**
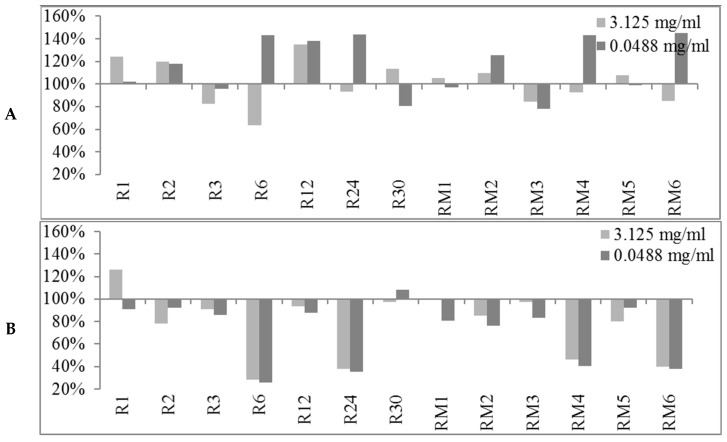

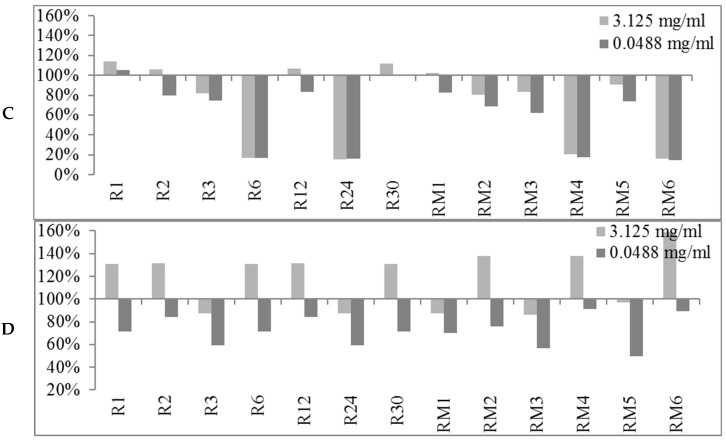
Percentages of biofilm production after treatment at two concentrations (3.125 mg/mL and 0.0488 mg/mL) for RSEOs against the four strains *S. aureus* 6538P (**A**) and 25923 (**B**), *S. epidermidis* RP62A (**C**) and O-47 (**D**, respectively). In the ordinate axis are reported the percentage of bacterial biofilm production. Data are reported as percentage of residual biofilm after the treatment in comparison with the untreated one. Each data point is composed of 4 independent experiments each performed with at least three replicates.

**Figure 2:**
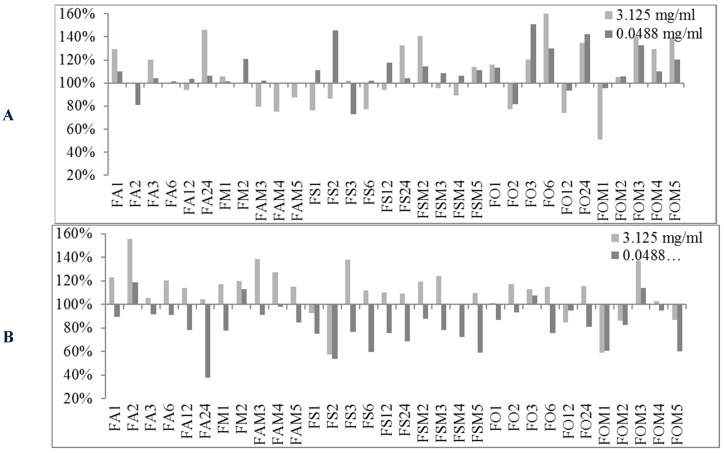

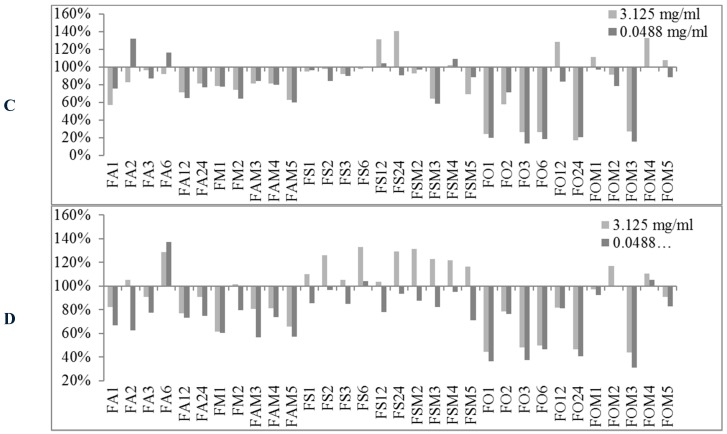
Percentages of biofilm production after treatment at two concentrations (3.125 mg/mL and 0.0488 mg/mL) for FVEOs against the four strains *S. aureus* 6538P (**A**) and 25923 (**B**), *S. epidermidis* RP62A (**C**) and O-47 (**D**, respectively). In the ordinate axis are is reported the percentage of bacterial biofilm production. Data are reported as percentage of residual biofilm after the treatment in comparison with the untreated one. Each data point is composed of 4 independent experiments each performed with at least three replicates.

**Figure 3:**
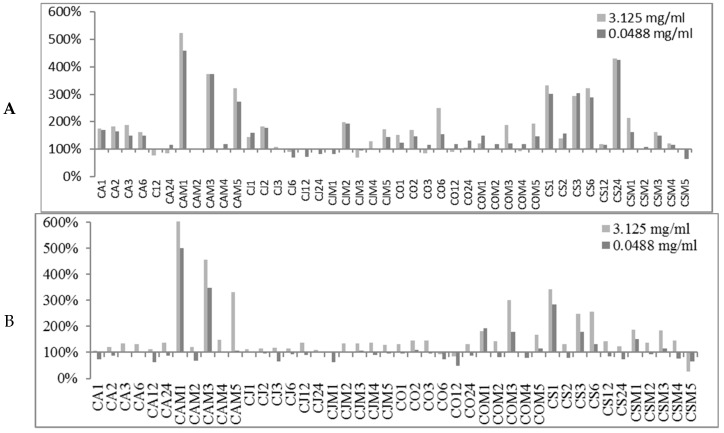

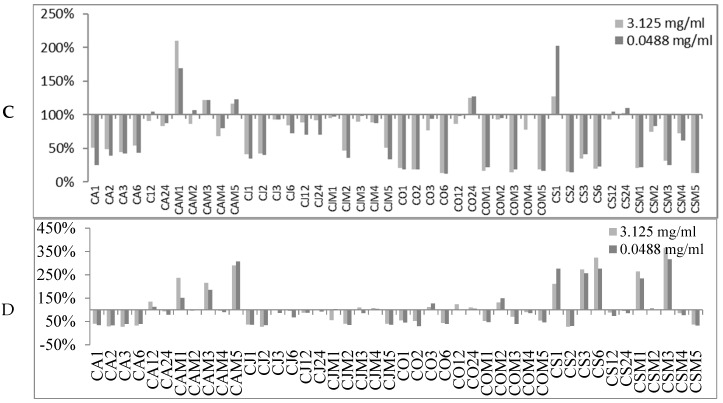
Percentages of biofilm production after treatment at two concentrations (3.125 mg/mL and 0.0488 mg/mL) for CGEOs against the four strains *S. aureus* 6538P (**A**) and 25923 (**B**), *S. epidermidis* RP62A (**C**) and O-47 (**D**), respectively). In the ordinate axis are is reported the percentage of bacterial biofilm production. The abscissa axis is centered at 100% biofilm production. Data are reported as percentage of residual biofilm after the treatment in comparison with the untreated one. Each data point is composed of 4 independent experiments each performed with at least three replicates.

The obtained preliminary data analyzed in terms of biofilm production modulation and reproducibility led to the selection of two representatives concentrations (3.125 mg/mL and 0.0488 mg/mL). The first concentration was in the range of milligrams while the second one was in the range of micrograms. All 89 EOs were then tested at the two selected concentrations and the biofilm production was measured relatively to untreated bacteria (Figure 1, Figure 2 and Figure 3). 

At either selected concentrations EOs modulated the biofilm production with unpredictable results for each strain. These results anticipated that many EOs may act mainly as biofilm inhibitors in the case of RP62A and O-47 strains, while for 6538P and 25923 EOs can either induce no effect or stimulate biofilm production (Table 4). In Table 4, the number of EOs able to inhibit (<100%, <80% and <50%, respectively) or stimulate (≥100%, ≥120%, ≥150% and ≥200%, respectively) biofilm formation is reported. It is worthy to note that on *S. epidermidis* strains about 30 EOs inhibited more than 50% of biofilm growth even at lowest concentration, while almost none of them showed an activity on *S. aureus* strains.

### 2.3. Quantitative Analysis of Selected EOs against Different Strains of S. epidermidis

**Figure 4:**
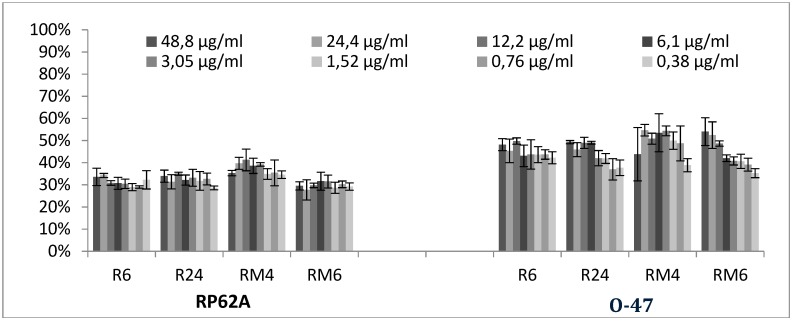
Antibiofilm effect of selected RSEOs on RP62A and on O-47 strains. In the ordinate axis is reported the percentage of bacterial biofilm production. Data are reported as percentage of residual biofilm after the treatment in comparison with the untreated one. Each data point is composed of 4 independent experiments each performed with at least in three replicates.

**Figure 5:**
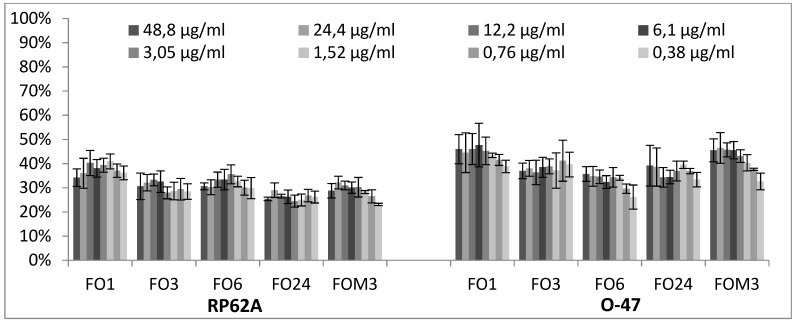
Antibiofilm effect of selected FVEOs on RP62A and on O-47 strains. In the ordinate axis is reported the percentage of bacterial biofilm production. Data are reported as percentage of residual biofilm after the treatment in comparison with the untreated one. Each data point is composed of 4 independent experiments each performed with at least three replicates.

**Figure 6:**
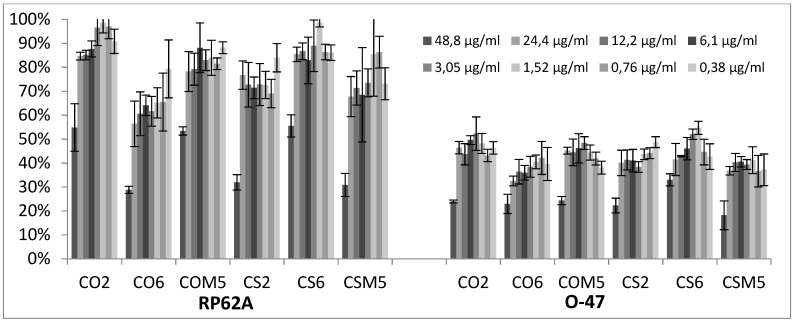
Antibiofilm effect of selected CGEOs on RP62A and on O-47 strains. In the ordinate axis is reported the percentage of bacterial biofilm production. Data are reported as percentage of residual biofilm after the treatment in comparison with the untreated one. Each data point is composed of 4 independent experiments each performed with at least three replicates.

Representative EOs selected among those able to reduce more than 70% of biofilm formation were further analyzed to evaluate a dose-dependent effect against *S. epidermidis* RP62A and O-47 (Figure 4, Figure 5 and Figure 6). The inhibition by RSEOs was confirmed at lower concentrations on both strains despite their different biofilm matrix composition and the inhibition of biofilm formation was clearly not dose-dependent (Figure 4). Analogous results were obtained with FVEOs samples (Figure 5). 

Differently, CGEOs revealed a dose dependent biofilm inhibition being more pronounced on the strongest biofilm producer *S. epidermidis* O-47 than on *S. epidermidis* RP62A (Figure 6). 

### 2.4. Application of Machine Learning Algorithms

#### 2.4.1. PCA Analysis of Datasets

Extraction of the first 3 PCs afforded to a cumulative explained variance of almost 90% (PC1:61.18%; PC1 + PC2: 75.02%; PC1 + PC2 + PC3: 82.92%). As reported [22], the first two principal components indicate at least three clusters (Supplementary Material Figure SM-1) and correctly identified the three plants derived EOs, although no definite separation between RSEOs and FVEOs was observable. PCA related loading plots indicated estragole, o-cymene and pulegone as the chemical components mainly related to high PCs values (Supplementary Material Figure SM-1). 

#### 2.4.2. Binary Classification Models

##### General Results

Analogously as in ML application to *Pseudomonas aeruginosa* (PA) [22] direct application of linear classification methods using algorithms such as Logistic Regression (LR) and Linear Support Vector Machines (SVM) [25] did not lead to satisfying classifiers (data not shown). At the same time non-linear algorithms like random forest (RF) [26], non-linear support vector machine (SVM) [27] and gradient boosting (GB) [28] also led to insufficiently robust models (data not shown). Therefore, a mixed approach was used and taking the idea from the principal component regression (PCR) as an evolution of multiple linear regression (MLR) a number of PCs were used in place of the original variables (EOs chemical component percentages) as input for the sklearn LR implementation (PCLR). As an initial test, the PCLR was run on the PA dataset leading to highly overlapping results with those obtained with the GB application (data not shown). Nevertheless, as biofilm production assay profiled EOs as either inhibitors of activators (Table 4) accordingly, classification models were tentatively built for all four strains considering either biofilm production inhibition or activation for biofilm percentages observed at the two above introduced concentration levels of 48.8 μg/mL and 3.125 mg/mL. To this aim, initially the optimal biofilm production percentage cutoff for the binary classification was explored by systematically either decreasing it from a starting 80% to 60% or increasing from 120% to 140% for the inhibition or activation models, respectively, being the ranges arbitrarily chosen on the basis of Table 4 filled data. The models’ accuracy was monitored by the MCC value obtained by leave-one-out cross-validation. Following this protocol, for the inhibition models EOs samples characterized by higher values than the best performing cutoff of biofilm production percentage were classified as inactive, while those with lower values were considered active. On the contrary, regarding the biofilm production enhancer models, EOs samples characterized by higher percentages than the cutoff value were classified as active, while those with lower values were considered non-active. Regarding the 6538P inhibition training set, the very low active/inactive ratio at biofilm inhibition below 80% prevented any optimization. Therefore, the grid search analysis to the starting sixteen training sets (four strains by two series of models by two concentrations) afforded to seven optimized models for either concentrations (Table 5). Inspection of optimized models on both hyperparameters and cutoff values revealed for 25923/inhibition, RP62A/activation and O-47/activation sets composed of high unbalanced ratios of actives over non-actives and were hence not further analyzed. Comparing developed models for the two used EOs concentrations revealed 3.125 mg/mL level to lead to more reliable and robust models (Table 6). Based on the above preliminary data, subsequent results and analyses were only carried out on RP62A/inhibition, O-47/inhibition, 6538P/activation and 259237/activation models derived for biofilm modulation recorded at 3.125 mg/mL. This is in full agreement with the fact that EOs samples acted prevalently as reducer of biofilm production for RP62A and O-47 strains, while for 6538P and 25923 the biofilm production was mainly enhanced (Table 4). 

To assess either models’ fitness and robustness, their lack of chance correlation was checked by Y-scrambling procedure whose 100 runs of cross-validated scrambled set led to average, standard deviation, maximum and minimum values for Accuracy_Y-S_, MCC_Y-S_, Precision-Recall_Y-S_ and ROC-AUC_Y-S_ ROC-AUC coefficients always lower than non-cross-validated and cross-validated ones, therefore assessing validity of all final models.

##### Binary Classification Model for 6538P Biofilm Production Activation

**Figure 7:**
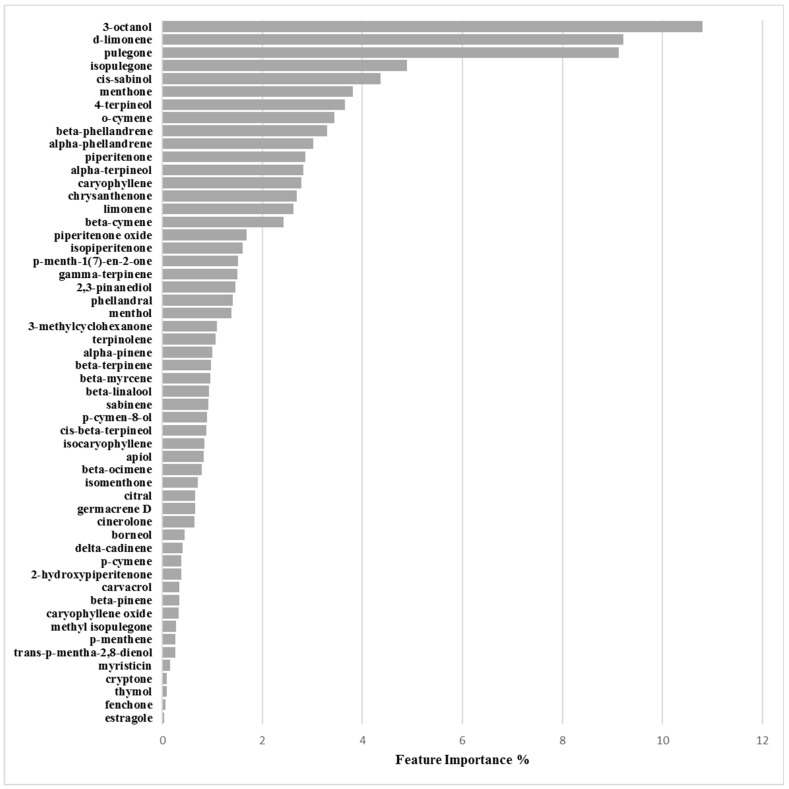
Feature importance plot for the 6538P/activation model defined at 3.125 mg/mL.

The 6538P/activation/3.125mg/mL optimized derived model was maximum at a cutoff of 133%, using 9 PCs, characterized by a 27:62 (0.44) proportion between actives and non-actives and high values of Accuracy (0.832), MCC (0.667), Precision-Recall (0.772) and ROC-AUC (0.824) coefficients which persisted to be quite good in cross-validation (Accuracy_CV_ = 0.805, MCC_CV_ = 0.613, Precision-Recall_CV_ = 0.698 and ROC-AUC_CV_ = 0.743) (Table 6 and Table 7). Feature importance and partial dependence pointed out compounds 3-octanol, d-limonene and pulegone as more important for biofilm production enhancement modulation (Figure 7, Supplementary Material Figure SM-1 and Table 8).

##### Binary Classification Model for RP62A biofilm production inhibition

The grid search on the EOs’ chemical composition and their associated RP62A biofilm production inhibitory potencies at 3.125 mg/mL, identified 62% biofilm residual production as the best cutoff value with only 5 PCs and a actives over non-actives ratio of 31:58 (0.53). The final classification model was found characterized by Accuracy, MCC, Precision-Recall and ROC-AUC values of 0.721, 0.455, 0.657 and 0.742, respectively (Table 6). Cross-validation associated coefficients Accuracy_CV_, MCC_CV_, Precision-Recall_CV_ and ROC-AUC_CV_ were 0.687, 0.392, 0.584 and 0.683, respectively.

**Figure 8:**
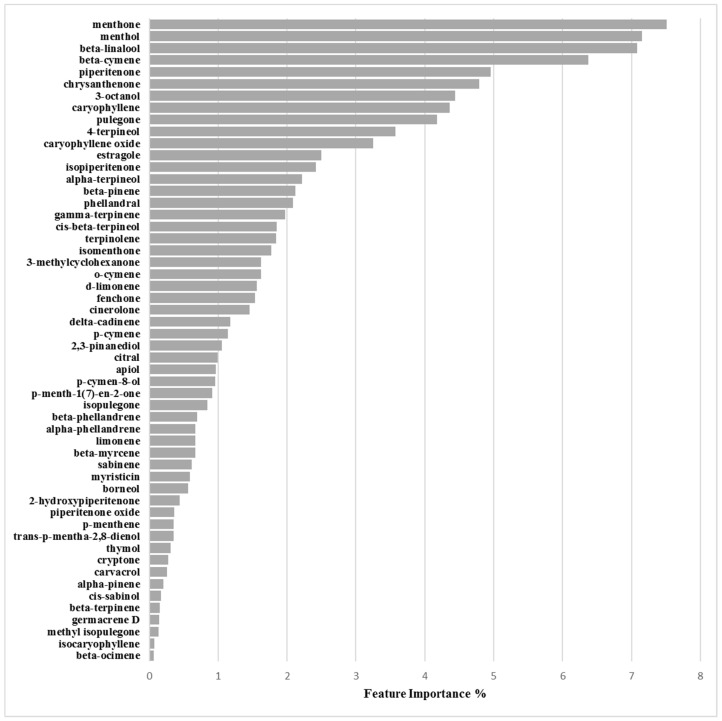
Feature importance plot for the 25923/activation model defined at 3.125 mg/mL.

Inspection of model associated EOs’ chemical components importance, the Skater algorithm indicated 3-octanol, phellandral, thymol and d-limonene as those mostly influencing biofilm production inhibition (Figure 8 and Table 7), whose positive control was highlighted by means of partial dependence plots which describe the marginal impact of a feature on model prediction (Supplementary Material Figure SM-2).

##### Binary Classification Model for O-47 Biofilm Production Inhibition

**Figure 9:**
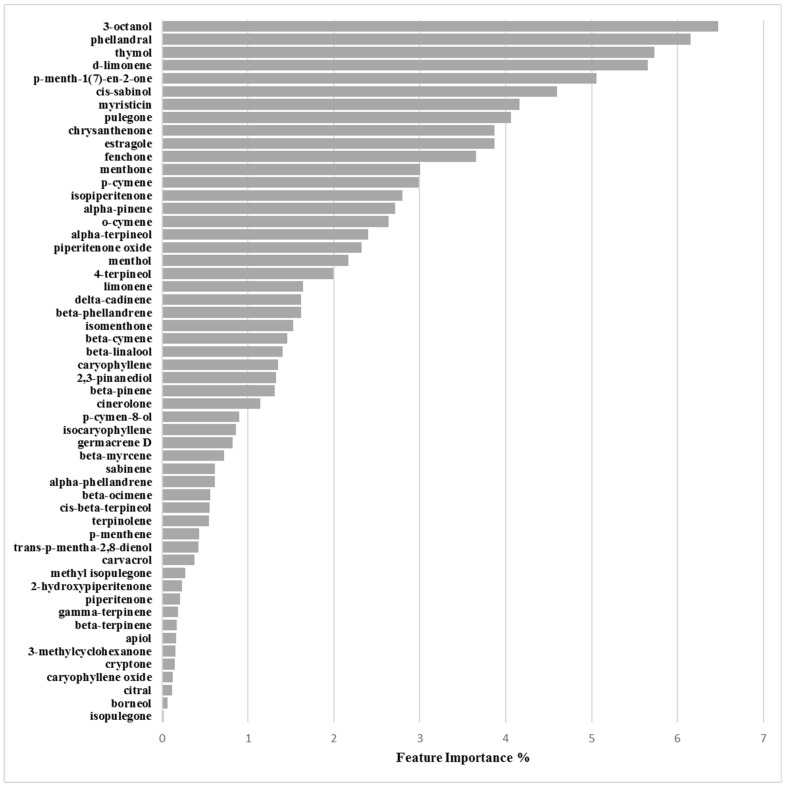
Feature importance plot for the RP62A/inhibition model defined at 3.125 mg/mL.

The O-47/inhibition/3.125mg/mL optimized derived model was also obtained at cutoff of 62%, but with 19 PCs and a 0.51 actives:non-actives ratio (30:59) leading to 0.771, 0.590, 0.682 and 0.753 values for the Accuracy, MCC, Precision-Recall and ROC-AUC coefficients, respectively. Model robustness was assessed by Accuracy_CV_, MCC_CV_, Precision-Recall_CV_ and ROC-AUC_CV_ values of 0.738, 0.517, 0.589 and 0.659, correspondingly (Table 6). Y-scrambling application did not revealed the presence of any chance correlation (Table 7). Inspection of feature importance and partial dependence pointed out as more significant for biofilm production inhibition the compounds 3-octanol, *o*-cymene, d-limonene and β-phellandrene (Figure 9, Supplementary Material Figure SM-3 and Table 8).

##### Binary Classification Model for 25923 Biofilm Production Activation

**Figure 10:**
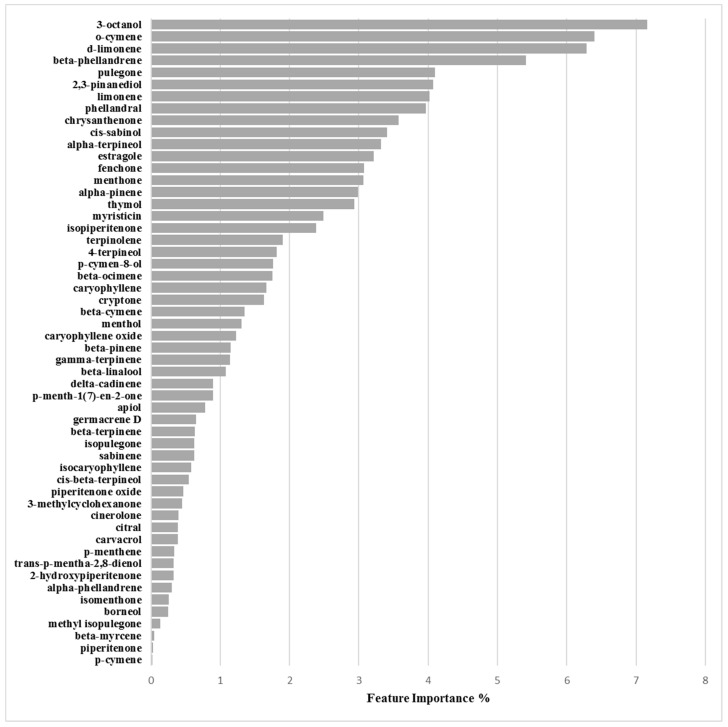
Feature importance plot for the O-47/inhibition model defined at 3.125 mg/mL.

The 25923/activation/3.125mg/mL optimized derived model was determined at cutoff of 121%, using 25 PCs, characterized by a 20:69 (0.29) proportion between actives and non-actives. The non cross-validated model was characterized by Accuracy, MCC, Precision-Recall and ROC-AUC coefficients of 0.906, 0.826, 0.956 and 0.961, respectively. Model robustness by cross-validation was characterized by high values of Accuracy_CV_ = 0.763, MCC_CV_ = 0.533, Precision-Recall_CV_ = 0.824 and ROC-AUC_CV_ = 0.834 (Table 6 and Table 7). Feature importance and partial dependence pointed out compounds d-limonene, γ-terpinene, 3-octanol and piperitenone as more important for biofilm production enhancement modulation (Figure 10, Supplementary Material Figure SM-4 and Table 8).

## 3. Discussion and Conclusions

### 3.1. EOs Biofilm Bioactivity General Consideration

From the results reported above it could be observed that each EO had a specific effect on biofilm formation, likely depending on its characteristics and unique chemical composition. In particular for *S. aureus* strains 6538P and 25923 the EOs mainly exhibited an enhancement of biofilm production. Stimulation of bacterial biofilm production by EOs is not surprising as it was previously observed, even by isolated chemical components [29,30,31]. On the other hand and more common [32,33], for *S. epidermidis* strains RP62A and O-47 an overall inhibition effect on biofilm production was observed by in vitro EOs treatment. Nevertheless, cinnamon EO was reported to stimulate biofilm production on some *Staphylococcus epidermidis* strains [34].

#### 3.1.1. Bioactivity of RSEOs

The majority of tested RSEO samples did not show inhibitory effects on *S. aureus* 6538P biofilm formation (a partial inhibitory effect was observed only for R6 essential oil at 3.125 mg/mL, panel A of Figure 1). On the contrary, some RSEO samples (R6, R12, R24, RM4 and RM6) were shown to enhance biofilm formation by up to 140% at 0.0488 mg/mL Differently, several RSEO samples showed a good inhibitory effect on *S. epidermids* RP62A biofilm production. In particular 4 out of 13 EOs (R6, R24, RM4 and RM6) were able to potently inhibit biofilm formation with a rate of about 80% at either used concentrations (panel B of Figure 1), thus these EOs were selected for further analyses using scalar concentration of each EO starting from 0.0488 mg/mL. An attempt to determine a direct dose dependent effect was not effective (Figure 4). 

On O-47 biofilm modulation (panel C of Figure 1) most RSEOs had a slight inhibition effect up to 40% (60% residual of biofilm production) at 0.0488 mg/mL. On the contrary, at the higher concentration RSEOs enhanced biofilm production by up to 130% for most samples. Only RM6 showed a remarkable biofilm production up to 160% at 3.125 mg/mL. For strain 25923 a profile similar to that of RP62A was observed (panel D of Figure 1), therefore no further investigation were pursued on the R6, R24, RM4 and RM6 samples despite the high inhibitory biofilm potency with residual biofilm production ranging 20–30%.

#### 3.1.2. Bioactivity of FVEOs 

Among all tested EOs, those from FV comprise among the most active samples able to inhibit biofilm production (Figure 2). In particular only mild effects (positive or negative modulation of biofilm production) were observed on 6538P strain (panel A of Figure 2) with a few exception at both selected concentrations, including FA24, FS2, FO3 and FO6 that increased biofilm production by about 40–60%, and FOM1 that inhibited biofilm production by about 50% at 3.125 mg/mL. On the contrary, some FVEOs proved to be potent antibiofilm agents on *S. epidermids* RP62A (panel B of Figure 2). In particular 5 out 33 FVEOs (FO1, FO3, FO6, FO24 and FOM3) inhibited biofilm formation with a rate of about 80% (panel B of Figure 2) and were selected for further analyses using scalar concentration of each EO starting from 0.0488 mg/mL. Similarly as for the selected potent RSEOs, no direct dose dependent effect was determined (Figure 5). Interestingly, on O-47 biofilm modulation most FVEOs showed a bioactivity profile almost overlapping that for RP62A with FO1, FO3, FO6, FO24 and FOM3 samples able to reduce biofilm formation of about 50–70% (panel C of Figure 2). While on both *S. epidermidis* strains RP62A and O-47, FVEOs displayed some peculiar samples with interesting biofilm inhibitory potencies in case of 25923 strain FVEOs displayed an overall bioactivity profile similar to that for RSEOs against O-47 (compare panel C of Figure 1 with panel D of Figure 2).

#### 3.1.3. Bioactivity of CGEOs 

EOs from CG are the most modulating biofilm producers either in positive (activators) or in negative (inhibitors). In particular in the case of strain 6538P all samples at either concentrations can be classified as neutral or biofilm promoters (panel A of Figure 3) with a strong inclination to increase biofilm production by up to 500% (CAM1). Other strong biofilm inducers (percentages over 300%) are CAM3, CAM5, CS1, CS3, CS6 and CS24 samples. Many other CGEOs, although to a lesser extent, induced a doubling or even tripling of biofilm production. On the contrary most of CGEOs displayed an inhibition by over 50% of biofilm production by RP62A (panel B of Figure 3). Many CGEO samples were further investigated and for 6 of them (CO2, CO6, COM5, CS2, CS6 and CSM5) a definite dose dependent relation was observed (Figure 6). Regarding biofilm modulation for O-47 CGEOs in this case presented, at either tested concentrations, a mixed scenario in which some samples induced an enhanced biofilm production up to 250-350% (CAM5, CS1, CS3, CS6, CSM1 and CSM3) and 15 different samples showed high inhibition potencies (percentages of residual biofilm lower than 40–50%). 

### 3.2. Machine Learning Classification Models

Application of the PCA coupled with logistic regression led to the formulation of 4 robust models that were characterized by quite good Accuracy, MCC, Precision-Recall and ROC-AUC values (Table 7). Model agnostic feature importance and partial dependence plots were used to find the marginal effect that each EO chemical component has on the predicted outcome of the binary classification models built on the 3.125 mg/mL response variables. Feature importance is a measure of the prediction error of the model after the feature’s values are permuted and highlights the absolute importance of each chemical constituent while partial dependence plots show whether the relationship between the bioactivity and the chemical component is linear, monotonous or more complex. 

#### 3.2.1. Biofilm Activation ML Model on 6538P

Inspection of feature importance for model derived on 6538P biofilm percentage production and EOs’ chemical compositions revealed 3-octanol, d-limonene and pulegone as the chemical components more associated to bacterial biofilm production (Figure 7 and Table 8). Further investigation of their partial dependence plots (Supplementary Material Figure SM-2) indicated those three chemicals as all positively correlated with biofilm enhancement. 

#### 3.2.2. Biofilm Activation ML Model on 25923

Similarly as for 6538P, also for the 25923 strain a ML model was built to correlate biofilm production enhancement with EOs’ chemical composition. Again, analysis of feature importance found as more important d-limonene, γ-terpinene, 3-octanol and piperitenone (Figure 8). Differently as found for 6538P the main component were not all positively associated to biofilm enhancement production, but d-limonene, γ-terpinene, 3-octanol were suggested to negatively modulate the increase of biofilm, while piperitenone was found positively correlated (Table 8 and Supplementary Material Figure SM-3)

#### 3.2.3. Biofilm Inhibition ML Model on RP62A

Differently from the previous model, feature importance associated to the EOs biofilm inhibition production on RP62A strain highlighted 3-octanol, phellandral, thymol, d-limonene as chemical compounds most important on modulating biofilm reduction (Figure 9 and Table 8). Partial dependence plots for 3-octanol, phellandral, thymol, d-limonene associated the four components as all positively able to inhibit biofilm production (Supplementary Material Figure SM-4).

#### 3.2.4. Biofilm Inhibition ML Model on O-47

Regarding ML model derived on the biofilm inhibition capability of EOs the compounds more responsible for biofilm production modulation were found to be 3-octanol, *o*-cymene, d-limonene and β-phellandrene (Figure 10 and Table 8). Differently from above RP62A analogous inhibition model only 3-octanol and d-limonene were found positively associated with EOs’ inhibitory ability by partial dependence plots (Supplementary Material Figure SM-5). On the contrary o-cymene and β-phellandrene were associated to a negative action on the inhibition. This could be speculated as a sort of anti-synergic effect that could balance EOs’ potencies.

#### 3.2.5. General Consideration on ML Models

According to the four classification models, two compounds, namely 3-octanol and d-limonene, can be considered as those that most influence biofilm production (Table 8). In particular, d-limonene positively correlated either in inhibiting or in enhancement of biofilm production in three out of the four models while has a negative modulation on the ML model built on the biofilm enhancement of EOs’ on 25923 strain. These data indicate some controversial mechanism associated to d-limonene, it could be speculated that being this compound a highly apolar monoterpene its role could not be indirectly associated to biofilm modulation by altering the bacterial wall [35] allowing other compounds, likely oxygenated ones to enter the cell acting in altering some biochemical mechanism that could end in stimulation or inhibition of biofilm production. Nevertheless, on this topic the data available in the literature is controversial: Natcha and Caoili [36] reported that d-limonene is effective in inhibiting the growth of S. epidermidis RP62A when combined with the antibiotic rifampicin, likely due to d-limonene interference with biofilm formation. The effect of d-limonene in inhibiting bacterial biofilm formation was also proved against species of the genus Streptococcus [37] for which minimal biofilm inhibitory concentration (MBIC) of 400 μg/mL was determined. In a very recent study d-limonene was also reported as a biofilm inhibitor, although less efficient than an EO containing d-limonene [38]. On the contrary Kerekes et al. assayed a series of EOs and a list of chemical components against food-related micro-organisms and found d-limonene was almost deprived of any ability to inhibit biofilm production. In a study from Espina et al. d-limonene at 2000 μL/L was reported to reduce the production of biofilm mass in *S. aureus* USA300 by 90% after 8 h of incubation, but increase it by 30% after 40 h of incubation [39]. EOs containing d-limonene and the isolated component were found to stimulate biofilm production on *Listeria monocytogenes* and antibiotic-resistant *Enterococcus faecalis* strains [29,30,31]. A similar profile and speculation on 3-octanol could also be deduced. 3-Octanol is a molecule resembling normal octanol, a compound commonly used to evaluate compound membrane permeability and lipophilicity through the determination of the logP parameter often used in ADME and QSAR studies. Unfortunately no data are available on the influence of 3-octanol on biofilm production, except for a single report in which the 8-carbon molecules 1-octen-3-ol, 3-octanol and 3-octanone specifically induced conidiation in Trichoderma species colonies placed in the dark [40]. Considering the possible cell wall permeation role of both d-limonene and 3-octanol for strains 6538P and 25923 pulegone, γ-terpinene and piperitenone, on the basis of the ML elaboration, could be the main components responsible for the modulation of EOs’ augmented biofilm production, while in the case RP62A and O-47 phellandral, thymol, *o*-cymene and β-phellandrene are mainly responsible for positively (phellandral and thymol) or negatively (*o*-cymene and β-phellandrene) modulating EOs’ biofilm inhibition. Unfortunately no specific data data are available on these isolated components and the herein discussion although based on robust ML calculation are not experimentally based. It is worthy to note that the four bacterial strains tested here produced biofilms with different characteristics. First 6538P and 25923 belong to *S. aureus* species, while RP62A and O47 belong to *S. epidermidis* species. 25923 is classified as a strong biofilm producer, and 6538P is a medium/strong biofilm producer according to Cafiso and coworkers [41]. Proteins are the major component in the biofilm matrix of 6538P, while in 25923 the polysaccharides have a predominant role. As regards the *S. epidermidis* strains, they are both strong biofilm makers and produce a biofilm mainly composed by polysaccharides. Moreover O-47 is a naturally occurring *agr* mutant [42]. As previously reported [43], *agr*-negative genotype enhanced biofilm formation on polymer surfaces by an increased expression of the surface protein AtlE, a bifunctional adhesin/autolysin abundant in the cell wall of *S. epidermidis*. The amount of AtlE present in the cell envelop is one of the reported differences between RP62A and O-47 [43]. The overexpression of AtlE could induce significant changes in the hydrophobicity of the bacterial surface [44]; this effect could explain the different action of EOs on these two strains. Furthermore, the classification models were developed on the same EOs tested on *P. aeruginosa* biofilm production [22]. In that case, investigation of the most important components by means of feature importance and partial dependence plots indicated estragole and phellandral as the chemical components mostly related to biofilm inhibition of *P. aeruginosa*, while d-limonene, pulegone and chrysanthenone seem to be related to its biofilm production. Although the use of feature importance and partial dependence plots shed some light on the possible role of some EOs’ components little is yet known on the role of the whole EOs mixture synergisms and anti-synergisms. Further studies on isolated EOs’ chemical components and on their simple mixture are currently under evaluation to develop more refined ML models able to disclose more details on the EOs’ mechanism of action.

## 4. Materials and Methods

### 4.1. Essential oil and Chemical Composition Analysis

EOs and their chemical compositions were available from previously reported studies [22,45,46]. Briefly, EOs were obtained by direct fractionated steam distillation and analyzed by a gas chromatographic/mass spectrometric (GC/MS) protocol [47,48].

### 4.2. Bacterial Strains and Culture Conditions

Bacterial strains used in this work (Table 9) were grown in Brain Heart Infusion broth (BHI, Oxoid, UK). Biofilm formation was assessed in static conditions. Planktonic cultures were grown in flasks under vigorous agitation (180 rpm) at 37 °C. In particular, *S. aureus* ATCC 6538P (6538P) and *S. aureus* ATCC 25923 (25923) are reference strains for antimicrobial testing; *S. epidermidis* RP62A (RP62A) is a reference strain isolated from infected catheter, while *S. epidermidis* O-47 (O-47) is a clinical isolate strong biofilm producer strain characterized by a genomic mutation in *agr locus* [42].

### 4.3. Determination of Minimal Inhibitory Concentration (MIC)

The MIC was determined as the lowest concentration at which the observable bacterial growth was inhibited. MICs were determined according to the guidelines of Clinical Laboratory Standards Institute (CLSI [49]). Each EO was added directly from mother stock and solutions were prepared by two-fold serial dilutions. Mother stock solutions were obtained by solubilizing each EO in DMSO at a final concentration of 1 g/mL. Appropriate dilution (10^6^ cfu/mL) of bacterial culture in exponential phase was used. Ten concentrations were used within the 25–0.045 mg/mL range. Experiments were performed in quadruplicate.

### 4.4. Biofilm Production Assay 

The quantification of biofilm production was based on microtiter plate biofilm assay (MTP): an opportune dilution of bacterial culture in exponential growth phase was added into wells of a sterile 96-well flat-bottomed polystyrene plate in absence and in presence of each EO. Quantification of in vitro biofilm production was based on previously reported methodology [50]. Briefly, the wells of a sterile 96-well flat-bottomed polystyrene plate were filled with 100 µL of the appropriate medium. 1/100 dilution of overnight bacterial cultures was added into each well (about 0.5 OD 600nm). As control, the first row contained bacteria grown in 100 μL of BHI (untreated bacteria). In the second row was added BHI supplemented with each EO at concentrations of 3.125 mg/mL and 0.0488 mg/mL, respectively. The plates were incubated aerobically for 18 h at 37 °C. Biofilm formation was measured using crystal violet staining. After treatment, planktonic cells were gently removed; each well was washed three times with double-distilled water and patted dry with a piece of paper towel in an inverted position. To quantify biofilm formation, each well was stained with 0.1% crystal violet and incubated for 15 min at room temperature, rinsed twice with double-distilled water, and thoroughly dried. The dye bound to adherent cells was solubilized with 20% (*v*/*v*) glacial acetic acid and 80% (*v*/*v*) ethanol. After 30 min of incubation at room temperature, OD590 was measured to quantify the total biomass of biofilm formed in each well. Each data point is composed of 4 independent experiments, each performed at least in 3-replicates. EOs altering biofilm formation of selected strains were then tested as reported below. Briefly, the wells of a sterile 96-well flat-bottomed polystyrene plate were filled with 100 µL of the appropriate medium. 1/100 dilution of overnight bacterial cultures was added into each well (about 0.5 OD 600nm). As control, the first row contained bacteria grown in 100 μL of BHI (untreated bacteria). Furthermore, BHI broth was added to remaining wells starting from the third row. In the second row was added BHI supplemented with each EO at a concentration of 0.0488 mg/mL. Starting from this lane, samples were serially diluted (1:2 dilutions). The plates were incubated aerobically for 18 h at 37 °C. Biofilm formation was measured using crystal violet staining, as previously reported. 

### 4.5. Statistical Analysis of Biological Evaluation

Data reported were statistically validated using Student’s t-test comparing mean absorbance of treated and untreated samples. The significance of differences between mean absorbance values was calculated using a two-tailed Student’s t-test. A *p* value of <0.05 was considered significant.

### 4.6. Machine Learning Binary Classification

#### 4.6.1. General Methods

All calculations were performed using the Python (version 3.6, https://www.python.org/) programming language [51] by executing in-house code in the Jupyter Notebook platform (version 5.7) [52]. The datasets were imported and loaded into a Pandas [53] dataframe and pre-processed to obtain four independent data matrices consisting of 89 rows (essential oil samples) and 54 columns (chemical components). Two dependent target vectors containing 89 biofilm production percentage observations at 48 μg/mL and 3.125 mg/mL were defined. Machine learning algorithms used in this study were implemented using the sklearn library (version 0.20) [54]. Unsupervised dimensionality reduction was performed with Principal component analysis (PCA) [55] while L2 regularized logistic regression was used for the supervised learning analysis. The scores and loadings relatives to the first two principal components (PCs) were graphically inspected on plots generated using the matplotlib library (version 3.0) [56]. To build the classification models, 30 PCs were extracted for each dataset. Cross-validation was used to search for the optimal inhibition/activation percentage cut-off values in order to define active and inactive samples. The optimal cut-off values were used to obtain the hyper-parameters optimized classification models. The Hyper-parameters optimization was achieved through a Bayesian optimization [57] of the number of PCs to be used as features and the regularization parameter of the L2-Logistic Regression (inverse of regularization strength in the sklearn implementation). For each dependent target vector two types of models were built: one to define EOs ability to inhibit biofilm production and another to describe biofilm production enhancement. Percentage ranges of 60–80% and 120–140% biofilm productions were chosen for inhibition and activation models, respectively. Finally, the most appropriate cut-offs for binary classification of biofilm inhibitors/not-inhibitors or biofilm enhancers/not-enhancers EOs were determined from a supervised learning analysis.

The binary classification models were numerically and graphically evaluated by accuracy (ACC), Matthews correlation coefficient (MCC), receiver operating characteristic (ROC) and precision-recall (PR) curves. Finally, the importance of EOs chemical components was evaluated individually through the “feature importance” and “partial dependence” plots [28] as implemented in the Skater python library [58,59]. Feature importance is a generic term for the degree to which a predictive model relies on a particular feature. Skater feature importance implementation is based on an information theoretic criteria, measuring the entropy in the change of predictions, given a perturbation of a given feature [58].

#### 4.6.2. Classification Models’ Validation

Validation of each classification model was carried out by leave-one-out cross-validation and taking into account the accuracy (ACC), the precision or positive predictive value (PPV), the recall or sensitivity or true positive rate (TPR), specificity or true negative rate (TNR), receiver operating characteristic (ROC) curve and the Matthews correlation coefficient (MCC) (see Supplementary Information) [22,60]. Y-scrambling [61,62] was ultimately applied to check any lack of chance correlation and assess coefficients robustness.

## Figures and Tables

**Table 1:** MIC determined for CGEOs on *S.* spp strains. EOID indicates samples names. Sample names are the same as previously reported [22]. Ofloxacin MIC is also reported as a positive reference drug. All data are expressed in mg/mL.

EOID	*S. aureus* 6538P	*S. aureus* 25923	*S. epidermidis* RP62A	*S. epidermidis* O-47
CJ1	>25	>25	>25	>25
CJ2	>25	>25	50	>25
CJ3	25	25	>25	>25
CJ6	25	25	>25	>25
CJ12	25	>25	>25	>25
CJ24	>25	>25	>25	>25
CJM1	12.5	25	>25	>25
CJM2	>25	>25	>25	>25
CJM3	12.5	25	25–12.5	25–12.5
CJM4	12.5	25	25	>25
CJM5	>25	>25	>25	>25
CA1	>25	>25	>25	>25
CA2	>25	>25	>25	>25
CA3	25	>25	>25	>25
CA6	25–12.5	>25	25	25
CA12	12.5	12.5	12.5	>25
CA24	12.5	12.5	>25	>25
CAM1	>25	>25	>25	>25
CAM2	12.5–6.25	12.5–6.25	12.5	12.5
CAM3	>25	>25	>25	>25
CAM4	6.25	6.25	12.5	12.5
CAM5	>25	>25	>25	>25
CS1	>25	>25	>25	>25
CS2	>25	>25	>25	>25
CS3	>25	>25	>25	>25
CS6	>25	>25	>25	>25
CS12	12.5–6.25	12.5	12.5	12.5
CS24	6.25	12.5–6.25	>25	12.5
CSM1	>25	>25	>25	>25
CSM2	12.5	12.5	12.5	12.5
CSM3	>25	>25	>25	>25
CSM4	12.5–6.25	12.5–6.25	12.5	12.5
CSM5	>25	>25	>25	>25
CO1	>25	>25	>25	>25
CO2	>25	>25	>25	>25
CO3	25–12.5	25	25	25
CO6	25–12.5	25–12.5	25–12.5	25–12.5
CO12	12.5	12.5	12.5	12.5
CO24	>25	>25	>25	>25
COM1	25	25	25–12.5	25–12.5
COM2	25–12.5	25–12.5	25–12.5	25–12.5
COM3	25	25	25–12.5	25–12.5
COM4	12.5	25	25	25
COM5	>25	>25	>25	>25
Ofloxacin	0.0002–0.0004	0.0004–0.0008	0.0002–0.0004	0.0002–0.0004

**Table 2:** MIC determined for FVEOs samples on *S.* spp strains. EOID indicates samples names. Sample names are the same as previously reported [22]. Ofloxacin MIC is also reported as a positive reference drug. All data are expressed in mg/mL.

EOID	*S. aureus* 6538P	*S. aureus* 25923	*S. epidermidis* RP62A	*S. epidermidis* O-47
FA1	>25	>25	>25	>25
FA2	25–12.5	25–12.5	>25	>25
FA3	>25	>25	>25	>25
FA6	>25	>25	>25	>25
FA12	>25	>25	>25	>25
FA24	12.5–6.25	>25	>25	>25
FAM1	>25	>25	>25	>25
FAM2	>25	>25	>25	>25
FAM3	>25	>25	>25	>25
FAM4	>25	>25	>25	>25
FAM5	>25	>25	>25	25
FS1	>25	>25	>25	>25
FS2	>25	>25	>25	>25
FS3	>25	>25	>25	>25
FS6	>25	>25	>25	>25
FS12	>25	>25	>25	>25
FS24	>25	>25	>25	>25
FSM1	>25	>25	>25	>25
FSM2	>25	>25	>25	>25
FSM3	12.5–6.25	>25	>25	>25
FSM4	>25	>25	>25	>25
FSM5	25–12.5	12.5	>25	>25
FO1	25	25	>25	>25
FO2	>25	>25	>25	>25
FO3	25	25	>25	>25
FO6	25	25	>25	>25
FO12	>25	>25	>25	>25
FO24	>25	>25	>25	>25
FOM1	>25	>25	>25	>25
FOM2	>25	>25	>25	>25
FOM3	25–12.5	12.5	>25	>25
FOM4	>25	>25	>25	>25
FOM5	>25	>25	>25	>25
Ofloxacin	0.0002–0.0004	0.0004–0.0008	0.0002–0.0004	0.0002–0.0004

**Table 3:** MIC determined for RSEOs samples on *S.* spp strains. EOID indicates samples names. Sample names are the same as previously reported [22]. Ofloxacin MIC is also reported as a positive reference drug. All data are expressed in mg/mL.

EOID	*S. aureus* 6538P	*S. aureus* 25923	*S. epidermidis* RP62A	*S. epidermidis* O-47
R1	>25	>25	>25	>25
R2	>25	>25	>25	>25
R3	>25	>25	>25	>25
R6	>25	>25	>25	>25
R12	>25	>25	>25	>25
R24	25	>25	>25	>25
R30	25	25	25	25
RM1	>25	>25	>25	>25
RM2	>25	>25	>25	>25
RM3	>25	>25	>25	>25
RM4	>25	>25	>25	>25
RM5	>25	>25	>25	>25
RM6	>25	>25	>25	>25
Ofloxacin	0.0002–0.0004	0.0004–0.0008	0.0002–0.0004	0.0002–0.0004

**Table 4:** Data analysis of biofilm production modulation by EOs at the two selected concentrations as reported in Figure 1, Figure 2 and Figure 3. In pale green background are depicted biofilm inhibition values, while in pale red background are highlighted biofilm enhanced data.

Conc. μg/mL	*S.* spp Strains	Biofilm Production %		Number EOs Samples at Biofilm Production %						
		MIN	MAX	<50%	<80%	<100%	≥100%	≥120%	≥150%	≥200%
3125	6538P	50.98	523.83	0	10	31	58	38	22	9
	25923	26.92	697.45	1	4	16	73	47	14	9
	RP62A	13.04	209.69	26	42	71	18	8	1	1
	O-47	27.12	289.88	24	35	61	28	14	4	4
48.8	6538P	62.80	459.46	0	5	20	69	37	17	7
	25923	37.91	501.01	3	34	67	22	10	7	3
	RP62A	11.79	202.57	29	48	74	15	6	2	1
	O-47	0.44	306.60	31	48	74	15	7	4	2

**Table 5:** Characteristics of the grid search optimized models.

Assayed Conc. (μg/mL)	Models’ Parameters	Biofilm Inhibition Models			Biofilm Activation Models			
		RP62A	O-47	25923	RP62A	O-47	6538P	25923
3125	PCs ^1^	5	19	22	9	12	9	25
	Actives ^2^	31	30	4	6	4	27	20
	Non-actives ^3^	58	59	85	83	85	62	69
	cutoff	62	62	63	126	133	139	139
48.8	PCs ^1^	8	9	24	15	8	9	20
	Actives ^2^	32	30	3	7	4	30	45
	Non-actives ^3^	57	59	86	82	85	59	44
	Cutoff^4^	63	63	62	124	138	133	121

^1^: number of principal components used in the model; ^2^: number of EOs as inhibitors or enhancers of bacterial biofilm production: ^3^: number of EOs as non-inhibitors or not-enhancers of biofilm production; ^4^: optimal values of bacterial biofilm production percentage for binary classification as inhibitors/non-inhibitors or enhancers/not-enhancers of bacterial biofilm production.

**Table 6:** Fitted and cross-validated Accuracy, MCC, Precision-Recall and ROC-AUC coefficients for the RP62A/inhibition, O-47/inhibition, 6538P/activation and 259237/activation optimized models at 3.125 mg/mL and 0.0488 μg/mL.

Validation	Assayed Conc. (μg/mL)	Coefficient	Biofilm Inhibition Models		Biofilm Activation Models	
			RP62A	O-47	6538P	25923
Fitting	3125	Accuracy	0.721	0.771	0.832	0.906
		MCC	0.455	0.590	0.667	0.826
		Precision-Recall	0.657	0.682	0.772	0.956
		ROC-AUC	0.742	0.753	0.824	0.961
	48.8	Accuracy	0.722	0.780	0.806	0.763
		MCC	0.452	0.604	0.632	0.533
		Precision-Recall	0.659	0.681	0.757	0.824
		ROC-AUC	0.735	0.752	0.815	0.834
Cross-Validation	3125	Accuracy_CV_	0.687	0.738	0.805	0.784
		MCC_CV_	0.392	0.517	0.613	0.568
		Precision-Recall_CV_	0.584	0.589	0.698	0.782
		ROC-AUC_CV_	0.683	0.659	0.743	0.845
	48.8	Accuracy_CV_	0.663	0.721	0.722	0.606
		MCC_CV_	0.335	0.474	0.450	0.214
		Precision-Recall_CV_	0.577	0.591	0.668	0.533
		ROC-AUC_CV_	0.666	0.660	0.753	0.599

**Table 7:** Chance correlation control by Y-scrambling procedure results. Mean, standard deviation (St Dev), maximum (max) and minimum (min) values for Accuracy_Y-S_, MCC_Y-S_, Precision-Recall_Y-S_ and ROC-AUC_Y-S_ ROC-AUC coefficients for cross-validated 100 runs. Values refer to RP62A/inhibition, O-47/inhibition, 6538P/activation and 259237/activation optimized models at 3.125 mg/mL.

Type of Model	Strain	Coefficient	Mean	St Dev	Max	Min
Biofilm Inhibition Models	RP62A	Accuracy_Y-S_	0.500	0.079	0.644	0.219
		MCC_Y-S_	0.000	0.159	0.290	−0.567
		Precision-Recall_Y-S_	0.496	0.063	0.643	0.353
		ROC-AUC_Y-S_	0.459	0.094	0.627	0.198
	O-47	Accuracy_Y-S_	0.494	0.081	0.665	0.286
		MCC_Y-S_	−0.011	0.164	0.342	−0.429
		Precision-Recall_Y-S_	0.506	0.068	0.668	0.377
		ROC-AUC_Y-S_	0.474	0.089	0.645	0.241
Biofilm Activation Models	6538P	Accuracy_Y-S_	0.492	0.082	0.637	0.249
		MCC_Y-S_	−0.017	0.169	0.275	−0.522
		Precision-Recall_Y-S_	0.507	0.071	0.661	0.347
		ROC-AUC_Y-S_	0.470	0.100	0.644	0.166
	25923	Accuracy_Y-S_	0.508	0.083	0.680	0.292
		MCC_Y-S_	0.013	0.170	0.361	−0.433
		Precision-Recall_Y-S_	0.524	0.071	0.746	0.355
		ROC-AUC_Y-S_	0.490	0.102	0.675	0.179

**Table 8:** Feature importances for each chemical component as derived by the SKATER algorithm for the RP62A/inhibition, O-47/inhibition, 6538P/activation and 259237/activation optimized models at 3.125 mg/mL. Background of more important chemical component for inhibition are colored in darker green, while in darker red background are highlighted components associated to higher biofilm production enhancement.

Chemical Component	Biofilm Inhibition Models		Biofilm Inhibition Models	
	RP62A	O-47	6538P	25923
2-Hydroxypiperitenone	0.23	0.32	0.38	0.44
2,3-Pinanediol	1.33	4.07	1.47	1.05
3-Methylcyclohexanone	0.16	0.45	1.09	1.62
3-Octanol	6.47	7.16	10.80	4.44
4-Terpineol	1.99	1.81	3.65	3.57
α-Phellandrene	0.61	0.30	3.02	0.67
α-Pinene	2.71	2.98	1.00	0.21
α-Terpineol	2.40	3.32	2.81	2.22
Apiol	0.17	0.78	0.83	0.97
β-Cymene	1.46	1.35	2.42	6.37
β-Linalool	1.40	1.08	0.93	7.08
β-Myrcene	0.72	0.05	0.95	0.67
β-Ocimene	0.56	1.75	0.78	0.06
β-Phellandrene	1.62	5.42	3.30	0.69
β-Pinene	1.31	1.15	0.34	2.12
β-Terpinene	0.17	0.63	0.97	0.15
Borneol	0.07	0.25	0.45	0.56
Carvacrol	0.38	0.38	0.34	0.26
Caryophyllene	1.35	1.67	2.78	4.36
Caryophyllene oxide	0.13	1.23	0.32	3.25
Chrysanthenone	3.87	3.58	2.68	4.79
Cinerolone	1.14	0.40	0.64	1.45
*cis*-β-Terpineol	0.55	0.55	0.88	1.85
*cis*-Sabinol	4.60	3.40	4.36	0.17
Citral	0.12	0.39	0.65	0.99
Cryptone	0.15	1.63	0.09	0.27
d-Limonene	5.66	6.29	9.22	1.56
delta-Cadinene	1.62	0.90	0.40	1.17
Estragole	3.87	3.21	0.04	2.49
Fenchone	3.66	3.08	0.06	1.54
γ-Terpinene	0.19	1.14	1.51	1.97
Germacrene D	0.82	0.65	0.65	0.14
Isocaryophyllene	0.86	0.58	0.84	0.07
Isomenthone	1.53	0.26	0.71	1.77
Isopiperitenone	2.80	2.38	1.61	2.42
Isopulegone	0.01	0.63	4.89	0.85
Limonene	1.64	4.02	2.62	0.67
Menthol	2.17	1.30	1.38	7.16
Menthone	3.00	3.07	3.81	7.51
Methyl isopulegone	0.27	0.14	0.27	0.13
Myristicin	4.16	2.48	0.16	0.59
*o*-Cymene	2.64	6.40	3.44	1.62
*p*-Cymen-8-ol	0.90	1.76	0.89	0.96
*p*-Cymene	2.99	0.02	0.39	1.14
*p*-Menth-1(7)-en-2-one	5.06	0.89	1.51	0.91
*p*-Menthene	0.43	0.33	0.26	0.35
Phellandral	6.15	3.97	1.40	2.08
Piperitenone	0.21	0.03	2.86	4.95
Piperitenone oxide	2.32	0.46	1.68	0.36
Pulegone	4.06	4.10	9.12	4.18
Sabinene	0.61	0.62	0.91	0.62
Terpinolene	0.55	1.90	1.07	1.84
Thymol	5.73	2.94	0.08	0.31
trans-*p*-Mentha-2,8-dienol	0.43	0.33	0.26	0.35

**Table 9:** Details of the used bacterial strains.

Strain	Name	Type	Isolation
*S. aureus* 6538P	6538P	clinical isolate	ATCC collection
*S. aureus* 25923	25923	clinical isolate	ATCC collection
*S. epidermidis* RP62A	RP62A	infected catheter isolated strain	ATCC collection
*S. epidermidis* O-47	O-47	septic arthritis clinical isolate	Heilmann et al., 1996 [29]

## Supplementary Materials

The following are available online. Figure SM-1: PCA first 2 PCs graphical plots. Scores plot (panel A) indicate the presence of at least three clusters (circled in panel A). Loading plots (panel B) highlights that estragole, *o*-cymene and pulegone could be the most important chemical constituents among all the tested EOs, Figure SM-2: 3-octanol, d-limonene and pulegone partial dependence plots for the activation model on 6538P biofilm production, Figure SM-3: d-limonene, γ-terpinene, 3-octanol and piperitenone partial dependence plots for the inhibition model on 25923 biofilm production, Figure SM-4: 3-octanol, phellandral, thymol and d-limonene partial dependence plots for the inhibition model on RP62A biofilm, Figure SM-5: 3-octanol, *o*-cymene, d-limonene and β-phellandrene partial dependence plots for the inhibition model on O-47 biofilm.
