# Supplementary material for: Machine Learning Analyses on Data including Essential Oil Chemical Composition and In Vitro Experimental Antibiofilm Activities against Staphylococcus Species
Source: Molecules. 2019 Mar 3;24(5):890. doi: 10.3390/molecules24050890 (PMC6429525; doi:10.3390/molecules24050890)
Supplement: Supplementary file 1 [file molecules-24-00890-s001.pdf]

molecules

Supplementary Material

Machine Learning Analyses on Data including Essential Oil Chemical Composition and In Vitro Experimental Antibiofilm Activities against *Staphylococcus* Species

Alexandros Patsilinakos [1,2,†], Marco Artini [3,†], Rosanna Papa [3], Manuela Sabatino [2], Mijat Božović [4], Stefania Garzoli [5], Gianluca Vrenna [3], Raissa Buzzi [6], Stefano Manfredini [6,*], Laura Selan [3,*] and Rino Ragno [1,2,*]

[1] Rome Center for Molecular Design, Department of Drug Chemistry and Technology, Sapienza University, P.le Aldo Moro 5, 00185 Rome, Italy; alexandros.patsilinakos@uniroma1.it
[2] Alchemical Dynamics s.r.l., 00125 Rome, Italy; manuela.sabatino@uniroma1.it
[3] Department of Public Health and Infectious Diseases, Sapienza University, P.le Aldo Moro 5, 00185 Rome, Italy; marco.artini@uniroma1.it (M.A.); rosanna.papa@uniroma1.it (R.P.); gianluca.vrenna@uniroma1.it (G.V.)
[4] Faculty of Natural Sciences and Mathematics, University of Montenegro, Podgorica, Montenegro; mijat.bozovic@uniroma1.it
[5] Department of Drug Chemistry and Technology, Sapienza University, P.le Aldo Moro 5, 00185 Rome, Italy; stefania.garzoli@uniroma1.it
[6] Master Course in Cosmetic Sciences, Department of Life Sciences and Biotechnology, University of Ferrara, Ferrara, Italy; raissa.buzzi@unife.it
* Correspondence: smanfred@unife.it (S.M.); laura.selan@uniroma1.it (L.S.); rino.ragno@uniroma1.it (R.R.), Tel.: +39-0532-455294 (S.M.); +39-6-4969-4298 (L.S.); +39-6-4991-3937 (R.R.); Fax: +39-0532-455294 (S.M.); +39-6-4969-4298 (L.S.); +39-6-4991-3627 (R.R.)
† These authors contributed equally to this work.

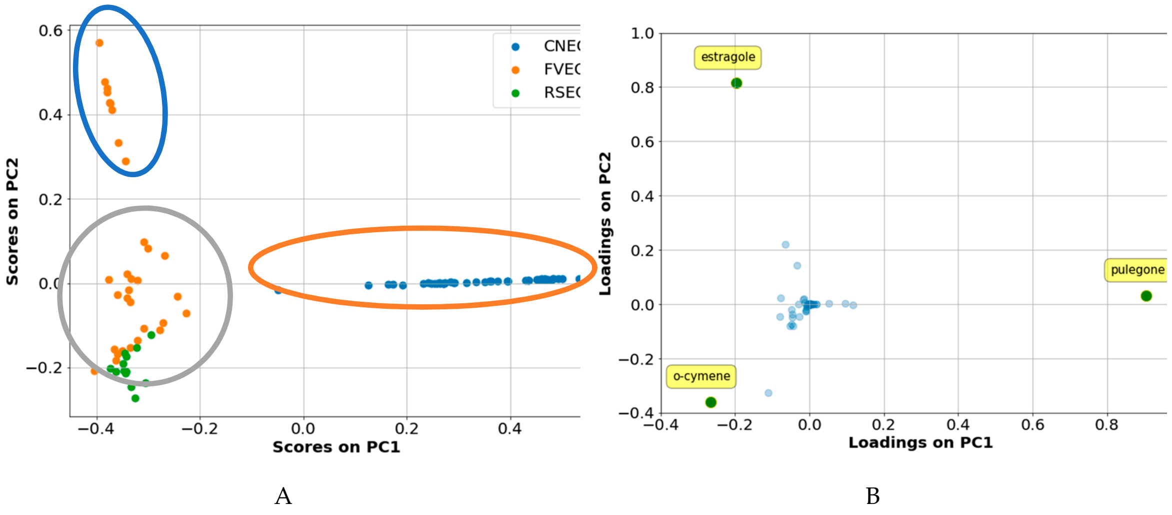

Figure SM-1. PCA first two PCs' graphical plots. Scores plot (panel A) indicate the presence of at least three clusters (circled in panel A). Loading plots (panel B) highlights that estragole, *o*-cymene and pulegone could be the most important chemical constituents among all the tested EOs.

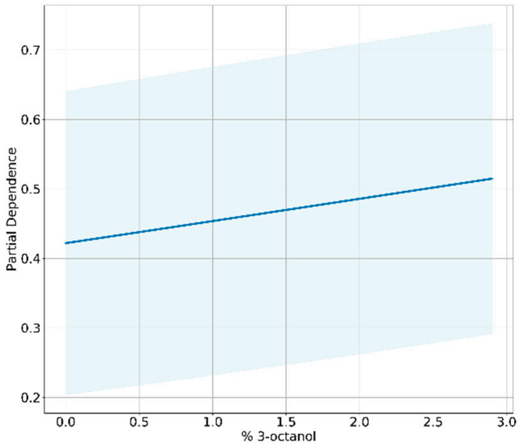

A

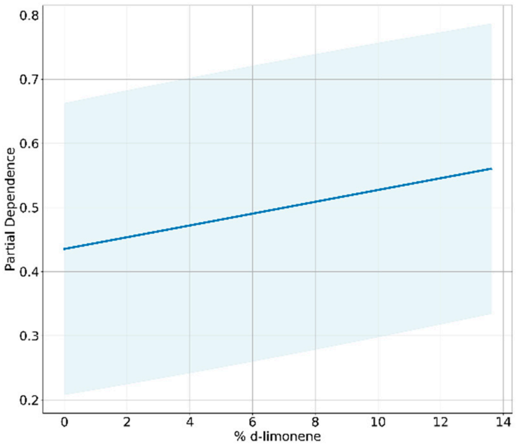

B

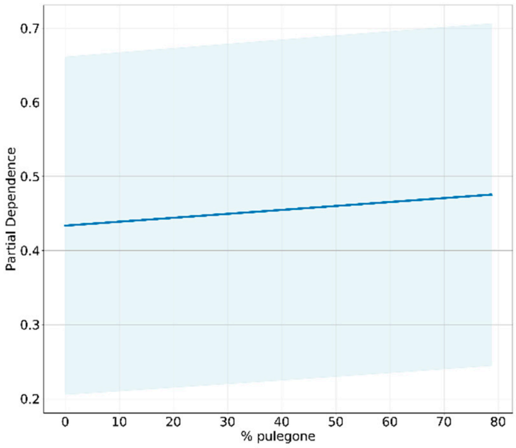

C

Figure SM-2. 3-octanol, D-limonene and pulegone partial dependence plots for the activation model on 6538P biofilm production.

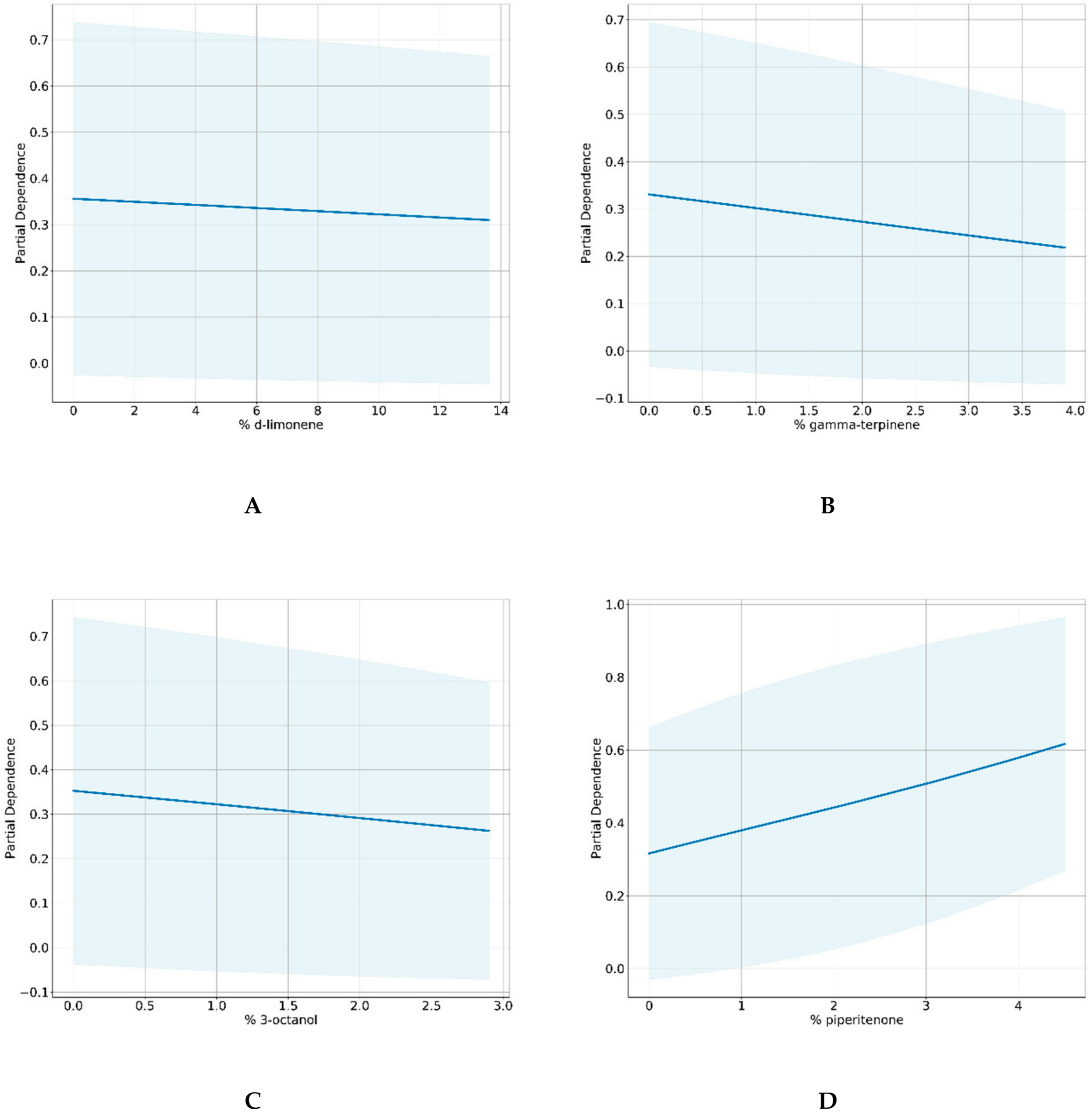

Figure SM-3. D-Limonene, γ-terpinene, 3-octanol and piperitenone partial dependence plots for the inhibition model on 25923 biofilm production.

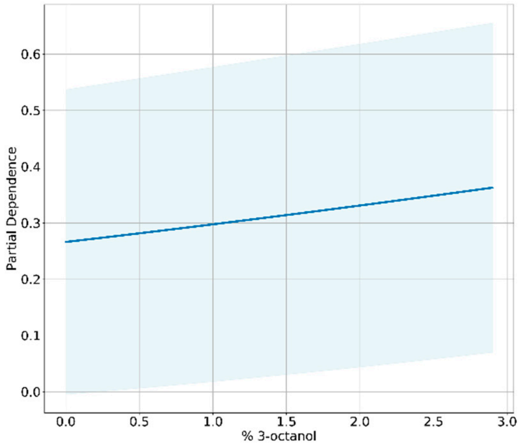

A

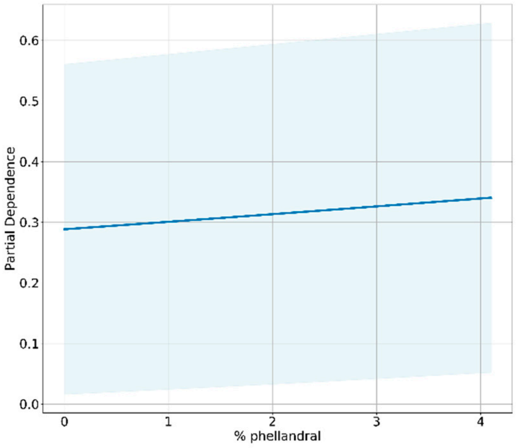

B

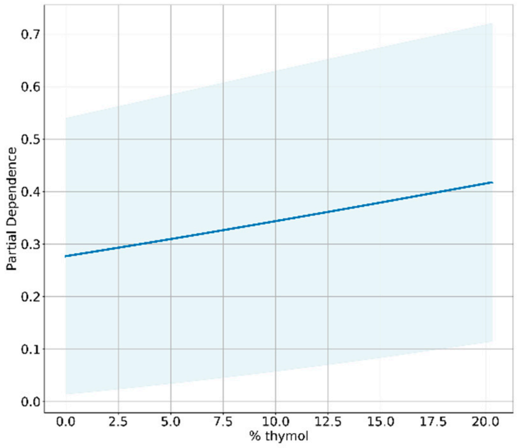

C

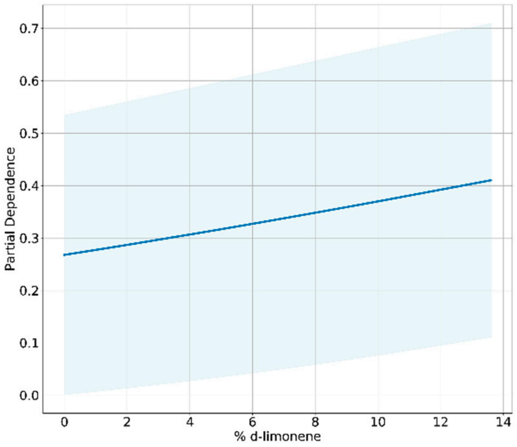

D

Figure SM-4. 3-Octanol, phellandral, thymol and D-limonene partial dependence plots for the inhibition model on RP62A biofilm.

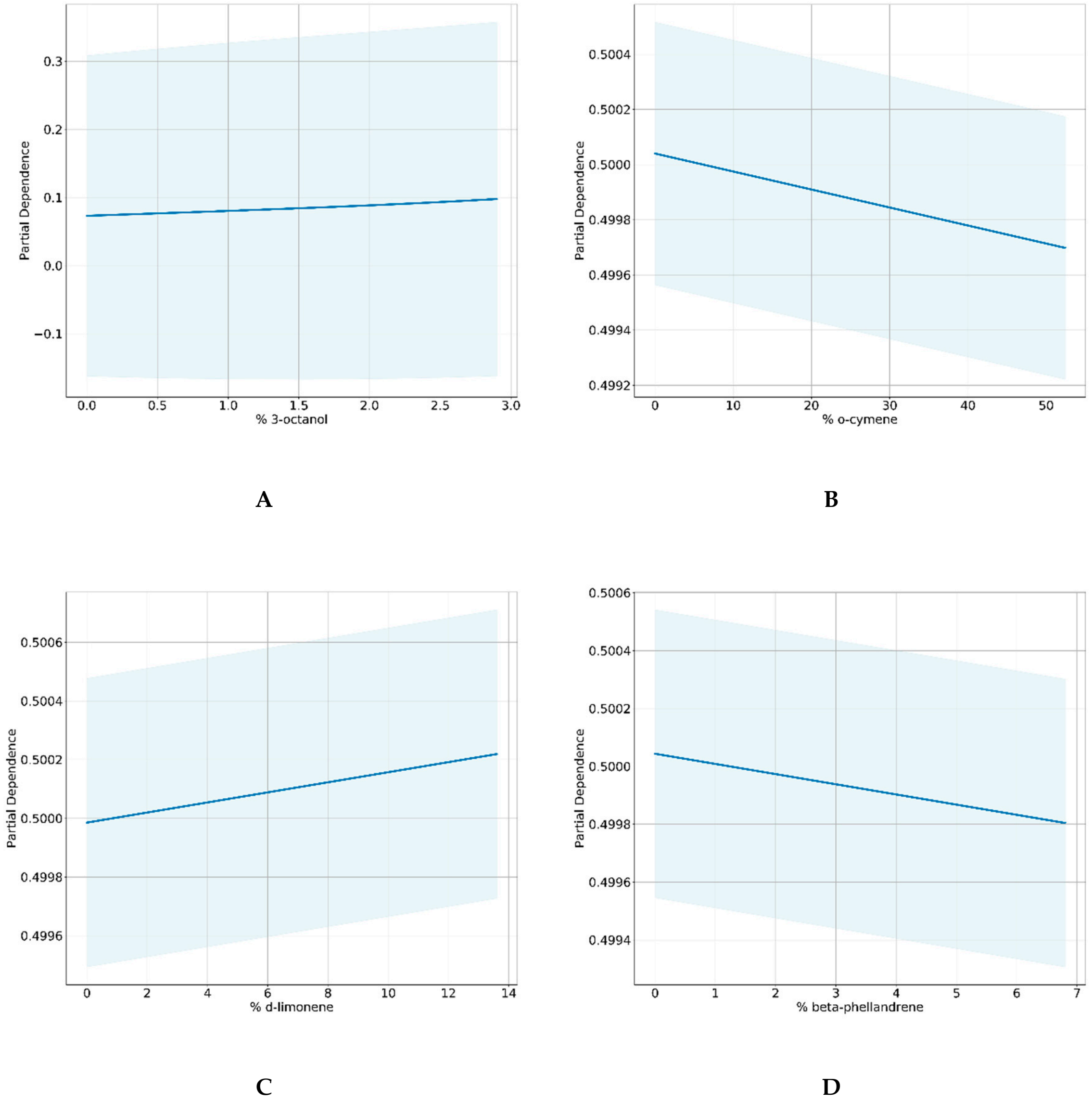

Figure SM-5. 3-Octanol, *o*-cymene, D-limonene and β-phellandrene partial dependence plots for the inhibition model on O-47 biofilm.
